# New Insights in CaVβ Subunits: Role in the Regulation of Gene Expression and Cellular Homeostasis

**DOI:** 10.3389/fcell.2022.880441

**Published:** 2022-04-06

**Authors:** Amélie Vergnol, Massiré Traoré, France Pietri-Rouxel, Sestina Falcone

**Affiliations:** INSERM U974, Center of Research in Myology-Sorbonne University, Paris, France

**Keywords:** CaVβs, CaV subunits, gene expression, calcium, cell homeostasis, diseases

## Abstract

The voltage-gated calcium channels (CaVs or VGCCs) are fundamental regulators of intracellular calcium homeostasis. When electrical activity induces their activation, the influx of calcium that they mediate or their interaction with intracellular players leads to changes in intracellular Ca^2+^ levels which regulate many processes such as contraction, secretion and gene expression, depending on the cell type. The essential component of the pore channel is the CaVα_1_ subunit. However, the fine-tuning of Ca^2+^-dependent signals is guaranteed by the modulatory role of the auxiliary subunits β, α_2_δ, and γ of the CaVs. In particular, four different CaVβ proteins (CaVβ1, CaVβ2, CaVβ3, and CaVβ4) are encoded by four different genes in mammalians, each of them displaying several splice variants. Some of these isoforms have been described in regulating CaVα_1_ docking and stability at the membrane and controlling the channel complex’s conformational changes. In addition, emerging evidences have highlighted other properties of the CaVβ subunits, independently of α_1_ and non-correlated to its channel or voltage sensing functions. This review summarizes the recent findings reporting novel roles of the auxiliary CaVβ subunits and in particular their direct or indirect implication in regulating gene expression in different cellular contexts.

## Introduction

Voltage-gated calcium channels (CaVs or VGCCs) are transmembrane ion channel proteins that act as major regulator of calcium-related cell functions. Their primary role is to mediate transmembrane calcium influx in response to membrane depolarization. Depending on their sensitivity to membrane depolarization, the activation of CaVs requires either a high or low threshold of membrane potential, dividing CaVs in High- and Low-voltage activated channels (HVA and LVA respectively) (Carbone and Lux, 1984; Fedulova et al., 1985). The crucial component of the channel pore is CaVα_1_, for which ten variants have been identified and classified based on their pharmacological properties and pore-opening kinetic: CaV1 and CaV2 for HVA, and CaV3 for LVA (Tsien et al., 1988; Catterall, 2011; Zamponi et al., 2015).

The CaV of skeletal muscle, also called dihydropyridine receptor (DHPR), was the first to be purified and cloned (Curtis and Catterall, 1984; Tanabe et al., 1987). In skeletal muscle fibers, CaV has a dual function of calcium channel and of voltage sensor of excitation-contraction coupling that controls, through a direct interaction, the opening of RyR1 (Ryanodine Receptor type 1), the Ca^2+^ release channel of the sarcoplasmic reticulum (Allard, 2018). Such a voltage sensor function for CaV and a direct interaction between CaV and RyR have also been described in neurons (Allard, 2018).

The main subunits of CaV, the αa1 subunits, are associated with auxiliary subunits that modulate expression and/or functional properties of the channel. In skeletal muscle, CaV1 is composed of five subunits: α1S (or CaV1.1), β1, α2δ, and γ (Hagiwara and Naka, 1964).

The function of both CaV1 and CaV2, members of HVA channels, needs the association of the auxiliary CaVβ subunit for their plasma membrane docking and proper gating (Schredelseker et al., 2005; Dayal et al., 2013), while the function of LVA class of channel (CaV3) is independent of this subunit (Zhang et al., 2013).

CaVβ are intracellular proteins that can either interact with the channel or be in their soluble form (Buraei and Yang, 2013).

Four different proteins, namely CaVβ1, CaVβ2, CaVβ3, and CaVβ4 encoded by four genes, exist in mammalians, each of them having several splice variants (Buraei and Yang, 2010). All CaVβ subunits are membrane-associated guanylate kinase (MAGUK) family members, with SH3 and GK domains as conserved domains whereas hook region, N- and C-terminal sequences are variables (Buraei and Yang, 2013). Hence, splice variants originate from alternative exon splicing and harbor different amino acidic compositions of variable regions, leading to specificities in protein interaction (Subramanyam et al., 2009; Obermair et al., 2010), subcellular targeting properties, and cellular localization, all influencing channel complexes stability, and activity (Campiglio et al., 2013).

Intracellular Ca^2+^ changes account for eukaryotic cell adaptation to external stimuli by modifying gene expression. By controlling Ca^2+^ influx into the cell, CaVs are therefore at the key position to mediate excitation-transcription (E-T) coupling. In point of fact, the mechanisms leading to CREB (cyclic AMP response element-binding) or NFAT (nuclear factor of activated T-cells) activation require the CaVs-dependent Ca^2+^ signaling (Dolmetsch et al., 2001; Hernández-Ochoa et al., 2007; Zhao et al., 2007). Noteworthy, if the role of CaVs in E-T coupling is mainly restricted to the initiation of the subsequent transcriptional activity of Ca^2+^, other mechanisms have been demonstrated to initiate gene regulation by generating a shorter isoform of the CaV1.2 pore forming subunit, which relocalized to the nucleus and held a transcription factor activity, (Gomez-Ospina et al., 2006; Gomez-Ospina et al., 2013). or by mobilizing intracellular CaVβ subunit after conformational changes upon membrane depolarization (Servili et al., 2018). Indeed, CaVβ2 subunit was recently demonstrated to be the mediator of CaV1.2-dependent E-T coupling, by interacting with H-Ras, which in turn activated MAPK (Mitogen Activated Protein Kinase)/ERK (Extracellular Signal-Regulated Kinase) pathway to induce CREB-directed gene expression in human neuronal SH-SY5Y cells (Servili et al., 2018). Nevertheless, the possibility that CaVs auxiliary subunits may be directly implicated as transcription factors has become an emerging hypothesis in the last 2 decades (Barbado et al., 2009).

This review will focus on CaVβs newly and less broadly described insights by illustrating their nuclei tracking in line with their role as regulators of gene expression.

## CaVβ as a Self-Sufficient Nuclear Protein

After the initial cloning of CaVβ1 in skeletal muscle [CaVβ1D formerly known as CaVβ1A (Traoré et al., 2019)] (Ruth et al., 1989), further works described several variants of CaVβ1 expressed in muscle and other tissues CaVβ1 (Hibino et al., 2003; Hullin et al., 2003; Foell et al., 2004; Harry et al., 2004; Cohen et al., 2005), lacking the domain required for its interaction with CaV*α*_1S_ at the α-Interaction Domain (AID domain). These reports suggested for the first time that some CaVβ subunits isoforms may have a CaV-independent function. Subsequently, several studies have demonstrated the capability of CaVβs to translocate to the nucleus, giving additional indications toward a role for CaVβ distinct from its well-known function as modulator of CaV channels.

If not all, at least some isoforms of CaVβ1, CaVβ2, CaVβ3 and CaVβ4 proteins display nuclear localization properties upon appropriate conditions (Buraei and Yang, 2010). For both CaVβ1, in skeletal muscle (Traoré et al., 2019), and CaVβ4, in neurons (Subramanyam et al., 2009; Etemad et al., 2014), this nuclear localization has been demonstrated to be linked to electrical activity. Indeed, our recent study showed that in adult skeletal muscle, after nerve damage, the embryonic isoform CaVβ1E was expressed and localized to the nuclei and near the Z-lines, while the constitutive adult muscle variant, CaVβ1D, remained associated with CaV1.1 at the sarcolemma (Traoré et al., 2019). Similarly, it has been reported that in neurons, CaVβ4A and CaVβ4B translocated to the nuclei when electrical activity was aborted, whereas CaVβ4E did not display nuclear localization. Additionally, a decrease in nuclear targeting of CaVβ1E (Traoré et al., 2019) and CaVβ4 (isoforms A and B) (Etemad et al., 2014) has been correlated with the onset of electrical activity throughout development in muscle fibers and neurons. Interestingly, the proportion of CaVβ4 isoforms targeted to nuclei has been associated with their activity as gene regulators (CaVβ4B > CaVβ4A > CaVβ4E) (Etemad et al., 2014). The mechanism originating the nuclear localization of CaVβ2 and CaVβ3 has not been clearly characterized, however, it can be hypothesized that they follow the same depolarization-sensitive process.

As previously mentioned, the capacity to get to the nucleus is not held by all the CaVβ1, CaVβ2, CaVβ3 and CaVβ4 splicing variants. The mechanisms underlying the specificities of the nucleus-targeted proteins could be passive diffusion through nuclear membrane for small proteins, while large ones need a Nuclear Localization Sequence (NLS), allowing their binding to Importins, or require the association with nuclear proteins as a shuttle. The molecular aspects behind CaVβs translocation to the nucleus are still not fully understood. For CaVβ1 (Buraei and Yang, 2010; Taylor et al., 2014) and CaVβ4 (Tadmouri et al. 2012) the SH3 domain of the protein has been described to exhibit the functional features leading to nuclear shuttling. An additional aspect was highlighted for CaVβ1E which have been described to display a putative NLS signal in its sequence (Taylor et al., 2014; Traoré et al., 2019), suggesting that its nuclear targeting was occurring through its binding to Importin proteins. However, modified genetic constructions lacking the putative NLS sequence did not prevent CaVβ1 to enter the nucleus. As an example, Subramanyam and colleagues showed that a specific double-arginine motif at the N-terminal was necessary and sufficient to induce the recruitment of the CaVβ4B variant toward the nuclei in mouse brain (Subramanyam et al., 2009). Nevertheless, this domain was subsequently demonstrated to be only partially involved in CaVβ4B docking to the nucleus, and that SH3/GK protein interaction domain was required to control its nuclear targeting. If these data were confirmed by several studies, a supplemental and non-exclusive mechanism came up with the demonstration of a PxxP binding motif in the SH3 domain of CaVβ1, raising the possibility that CaVβ proteins might also bind to proteins that themselves shuttle to the nucleus (Buraei and Yang, 2010). An instance supporting this hypothesis is CaVβ4C, which interaction with HP1γ has been shown as mandatory to localize to the nucleus in mammalian cells (Hibino et al., 2003), while the truncation of a large part of its GK domain cut off the exclusive requirement of SH3/GK interaction for nuclear docking of CaVβs proteins. The molecular aspects of nuclear targeting were less studied for CaVβ2 and CaVβ3, for which some studies mentioning their binding with chaperone proteins may be relevant in supporting their tackling to the nuclei (Zhang et al., 2010; Pickel et al., 2021).

The evidence of the nuclear localization/translocation of several CaVβ variants spotted these proteins with an undeniable CaV*α*_1_ independent function and pinpointed their putative role in the modulation of gene expression. The next part of this review will summarize the mechanisms described for the CaVβ auxiliary subunits in the regulation of gene transcription.

## CaVβs as Factors Controlling Gene Expression

### CaV-Independent Role of CaVβ in Calcium-Mediated Gene Expression

**FIGURE 1:**
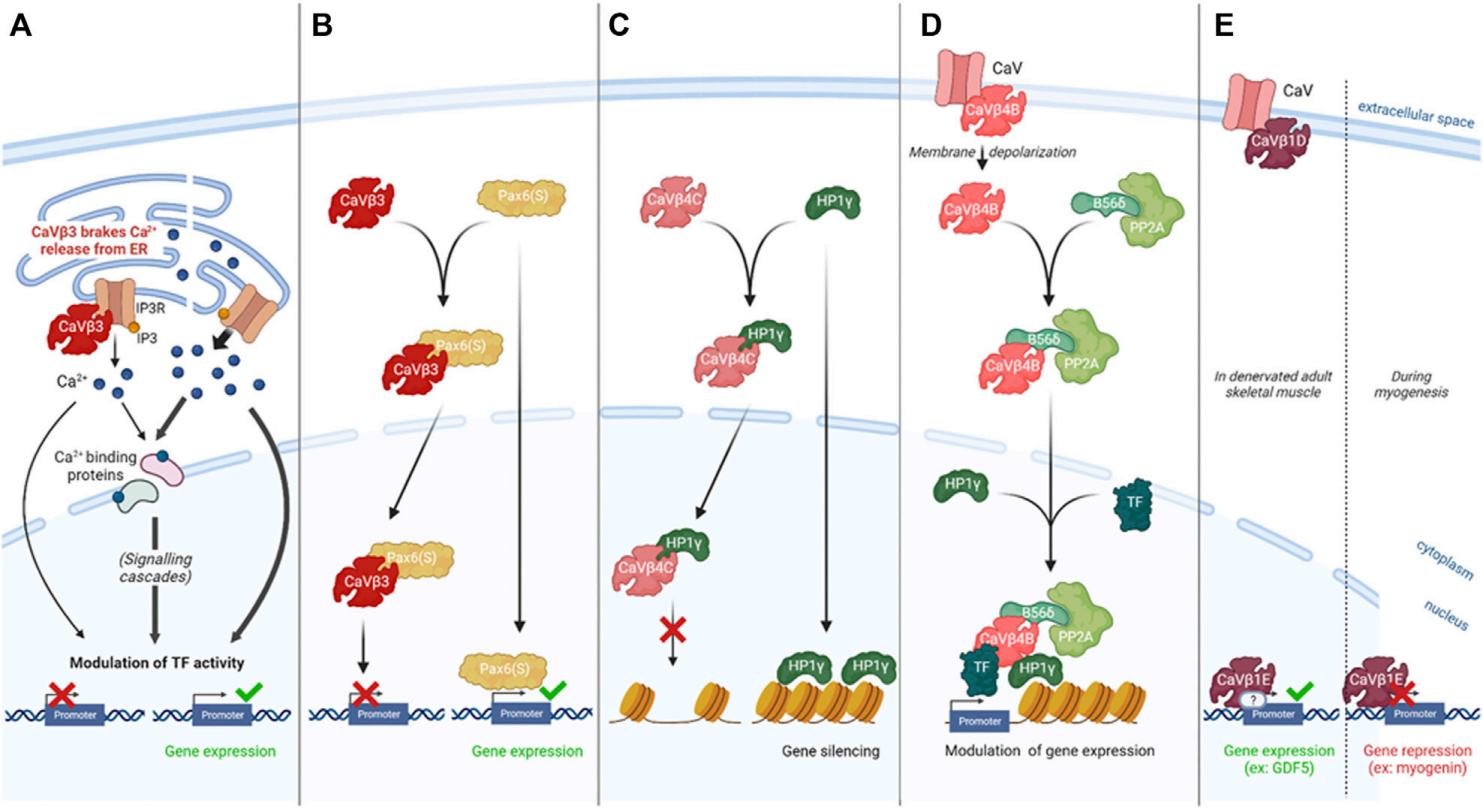
CaV-independent CaVβs functions in regulating gene expression.**(A)**. CaVβ3 interacts with IP3R to desensitize cells to low IP3 concentration and brake Ca2+ release, consequently interfering with the Ca^2+^-related modulation of gene expression and affecting glucose-triggered insulin secretion in β-pancreatic cells (Becker, et al 2021) and fibroblasts mobility (Belkacemi et al., 2018). **(B)**. CaVβ3 translocates to the nucleus with Pax6(S), preventing its transcriptional activity (demonstrated in a reporter assay *in vitro* in HEK 293T cells) (Zhang et al. 2010). **(C)**. CaVβ4C translocates to the nucleus with HP1γ, a factor known to silence the transcription of several genes by modulating heterochromatin conformation. CaVβ4C interaction with HP1γ prevents its gene silencing activity in mammalian cells (Hibino et al. 2003, Xu et al. 2011). **(D)**. CaVβ4B acts as an organizing platform for transcription-modulating factors including PP2A, HP1γ and the transcription factor TRα. CaVβ4B stabilizes this protein complex, allowing its modulatory activity on gene expression in hippocampal neurons in culture (Tadmouri et al. 2012). **(E)**. CaVβ1E acts directly or not on DNA regulatory sequences to modulate gene expression and influence myogenesis or skeletal muscle mass homeostasis after denervation (Taylor et al. 2014, Traoré et al. 2019).

As mentioned, a large set of intracellular processes are driven through Ca^2+^ signaling and therefore dependent on the free cytosolic calcium. Either CaVs-related Ca^2+^ entry from the extracellular space or mobilization of intracellular Ca^2+^ stock can modulate its cytosolic concentration and originate this signaling. As an auxiliary subunit, CaVβ has been described to regulate Ca^2+^ influx into the cell by modulating CaVs activity (Buraei and Yang, 2010), however, this protein was also reported to regulate intracellular Ca^2+^ in a CaV-independent way by acting on Ca^2+^ stores. Indeed, in both pancreatic cells (Berggren et al., 2004; Becker et al., 2021) and fibroblasts (Belkacemi et al., 2018), it has been demonstrated that CaVβ3 could interfere with Inositol 3-Phosphate (IP3)-induced Ca^2+^ release from the Endoplasmic Reticulum (ER) by binding IP3 Receptor (IP3R), therefore desensitizing cells to low IP3 concentration (Berggren et al., 2004; Belkacemi et al., 2018; Becker et al., 2021). In this process, CaVβ3 acted as a “brake” on Ca^2+^ release, affecting glucose-triggered insulin exocytosis in β-pancreatic cells (Berggren et al., 2004; Becker et al., 2021) and cellular mobility in fibroblasts (Belkacemi et al., 2018). These studies illustrated an effect of CaVβ subunits in affecting gene expression by regulating free cytosolic calcium concentration (Figure 1A)

### Interaction With Various Transcription Factors

CaVβ3 has been reported by Zhang and colleagues to co-localize with Pax6(S) in the nucleus and the interaction of these two proteins has been described to account for a ∼ 50% decrease in Pax6(S) transcriptional activity (demonstrated in *Xenopus* oocytes by reporter system *in vitro*) without impairing CaV channel properties (Zhang et al., 2010). More generally, Pax6 proteins are composed of two DNA-binding domains: a paired-domain (PD) and a homeodomain (HD), allowing the binding to the cis-elements of target genes to regulate their transcription rate, and a proline/serine/threonine (PST)-rich C-terminal domain, holding a trans-activation function. The work of Zhang and colleagues highlighted that Pax6(S) presented intact PD and HD domains while its C-terminal domain was truncated, resulting in a weaker Pax6(S) trans-activity. This isoform also differed from canonical Pax6 by a unique S-tail, originating its interaction with CaVβ3. This work suggested a novel function of CaVβ3 in negatively regulating Pax6(S) protein activity, although the precise mechanism, supposed to occur either by CaVβ3 allosteric hindrance or by Pax6(S) removal from DNA binding sites, remained undefined. More importantly, this report showed for the first time a full-length CaVβ protein having a role in the channel function, acting also directly as a modulator of gene transcription (Zhang et al., 2010) (Figure 1B).

In 2003, Hibino and colleagues identified from chicken cochlea the CaVβ4C variant, a truncated CaVβ4 isoform which is also expressed in the brain, eye, heart and lung, concomitantly with the full-length isoforms CaVβ4A and CaVβ4B. However, in contrast with these two isoforms, CaVβ4C was described to lack a large part of the GK domain necessary to associate with CaV*α*_1_ Chen et al., 2004, having therefore little effect on Ca^2+^ channel activity (Hibino et al., 2003). This -by then- newly identified variant showed a direct interaction with the chromo shadow domain (CSD) of the chromo box protein 2/heterochromatin protein 1γ (CHCB2/HP1γ), a nuclear protein that modulates the transcription of several genes by regulating heterochromatin conformation and therefore gene silencing. Noteworthy, the binding of HP1γ to DNA regions of euchromatin was shown to correlate with gene repression. Hibino’s study reported that when co-expressed with HP1γ, CaVβ4C was recruited to the nucleus, dramatically lowering the CHCB2/HP1γ gene-silencing activity *in vitro* (Hibino et al., 2003). This was the very first time that a CaVβ protein was described to translocate to the nucleus and act as a transcriptional regulator.

A few years later, the existence of CaVβ4C was revealed in the human brain and observed to also interact with the CSD of HP1γ (Xu et al., 2011). In addition, this interaction was shown to occur *via* a CSD binding motif, the PXVXL consensus sequence. Consistently with Hibino and colleagues’ work, the binding of human CaVβ4C to HP1γ was demonstrated to lead its nuclear translocation where it markedly reduces the gene-silencing activity of HP1γ *in vitro* (Xu et al., 2011). These studies illustrated a first manner for CaVβ subunits to indirectly modulate gene expression by affecting the activity of proteins involved in DNA compaction, like HP1γ (Figure 1C).

The first report of nuclear localization of the CaVβ4B full-length isoform has been achieved by Subramanyam and colleagues in neurons and muscle cells, where this nuclear localization was reported to negatively relate on electrical activity (Subramanyam et al., 2009). The comprehension of the localization-related role of this protein has been realized later, with the demonstration that it acted as an organizing platform of a group of proteins which controlled transcription (Tadmouri et al., 2012). Among the complex-forming proteins, B56δ, the regulatory subunit of the PP2A phosphatase induced histone dephosphorylation and HP1γ restructured heterochromatin. The last defined component of this complex was a transcription factor able to bind DNA at the promoter regions, allowing B56δ and HP1γ activity (Tadmouri et al., 2012). The gene that has been demonstrated to be modulated through this mechanism is Tyrosine hydroxylase (TH), the corresponding transcription factor being thyroid hormone receptor alpha (TRα) (Tadmouri et al., 2012). This situation was different from the previously described CaVβ4C effect on HP1γ activity, since CaVβ4B was, in this case, the element enabling the complex B56δ/HP1γ/TRα to access their activity site rather than modulating their function itself (Figure 1D). If Subramanyam and colleagues demonstrated a negative correlation between neuronal excitability and V5-tagged CaVβ4B positioning at the nucleus, this report showed that the endogenous CaVβ4B association with B56δ, originating their nucleus translocation, was consecutive to electrical activity, suggesting that the V5 tag might have hindered the pathways leading to CaVβ4B nuclear localization (Tadmouri et al., 2012).

Two further studies, aimed at investigating the property of CaVβ4 variants in controlling the expression of cell cycle-related genes, demonstrated that nuclear CaVβ4 full-length was able to inhibit cell proliferation, while its epileptogenic mutant, lacking C-term, had no impact. The effects of CaVβ4 on cell cycle were related to the ability of the CaVβ4 to interact to either B56δ or T-cell factor 4 (TCF4) transcription factors. In the first case, B56δ recruitment to the nuclei by CaVβ4 was suggested to mediate the repression of genes involved in cell proliferation (Rima et al., 2017a). On the other hand, the binding of CaVβ4 to TCF4 was demonstrated to prevent its interaction with β-catenin, as additional mechanism to inhibit the activation of β-catenin-Wnt-dependent gene expression and cell cycle (Rima et al., 2017b). These reports established the ability of a CaVβ isoform to control gene expression, autonomously from CaVs, either dependently on or independently of electrical activity.

### Interaction With Regulatory DNA Sequence

A study, published in 2014 by Taylor and colleagues, pinpointed that CaVβ1 was able to translocate to the nuclei and bind at the promoter region of 952 genes in muscle precursor cells (MPCs). Importantly, it showed that the absence of CaVβ1 resulted in changes in the expression of several genes, either positively or negatively misregulated, designating this subunit as a having a transcription factor function (Taylor et al., 2014). This role was more deeply confirmed for myogenin which was up-regulated in the absence of CaVβ1, preventing a correct myogenic development (Schuster-Gossler et al., 2007; Ho et al., 2011; Taylor et al., 2014). In this study, the CaVβ1A was the isoform described as a transcription factor, while our study published in 2019, rather indicated that the capacity to localize to the nucleus and exert a transcription factor role was actually carried by CaVβ1E which was identified as the main CaVβ1 isoform in C2C12 myoblast cell line, consistent with what observed in MPCs (Traoré et al., 2019). In addition, our work showed that the CaVβ1E played a crucial role in adult muscle mass homeostasis, when electrical activity was impaired, by regulating directly or indirectly the GDF5 promoter to trigger its transcriptional activity (Traoré et al., 2019) (Figure 1E).

By all these studies, CaVβs proteins have emerged as key players in regulating gene expression through Ca^2+^ signaling, DNA remodeling, modification of transcription factors activity or by acting as transcription factors themselves. When these functions are lost, multiple cellular functions are disturbed, involving CaVβ proteins in pathological conditions independently of the CaV-related aspect.

## Implication of CaVβs in Pathological Conditions

Although isoforms of CaVβ1, CaVβ2, CaVβ3, and CaVβ4 are expressed in several regions of the brain (Buraei and Yang, 2013), ablation of CaVβ1, CaVβ2 and CaVβ3 have no major impact on neuronal function (Ball et al., 2002). On the contrary, the relevance of CaVβ4 in the nervous system physiology was shown in the *lethargic (lh)* mouse model, having ataxic and epileptic phenotype (Burgess et al., 1997). Its implication in the pathophysiology of neuronal disorders was confirmed in Humans, after the discovery that missense and coding mutations, affecting the N-terminal region of the protein, were associated with epilepsy and ataxia, respectively (Escayg et al., 2000; Tadmouri et al., 2012). In the cerebellum, CaVβ4 is the most expressed and the major auxiliary subunit, together with α2δ-2, of the CaV2.1 calcium channel. Interestingly, mutations in all three proteins have been reported to lead to an epileptic and ataxic phenotype. Therefore, CaVβ4 involvement in such pathological conditions was first linked with its CaV-associated role (Escayg et al., 2000).

However, an additional mechanism to further elucidate such pathologies came from the CaV-independent role of CaVβ4. Indeed, at the molecular level, human epilepsy and ataxia-associated mutations were found to prevent CaVβ4 to shuttle toward nuclei by disrupting the SH3/GK domains interaction and indicated that mis-regulated CaVβ4-dependent gene transcription may have a key relevance in the pathophysiology of these neurological disorders (Tadmouri et al., 2012) (Table 1).

**TABLE 1:** CaVβs associated disorders.

	Pathology/Pathological features	CACNB gene	References–CaVβ in the pathological context	
			Description	CaV-independant disorders
Heart	Brugada Syndrome (BrS), Type 4	CACNB2 (causal mutation)	Carbone and Lux (1984); Fedulova et al. (1985)	—
	Hypertrophic cardiomyopathy (HCM)	CACNB2 (gene modifier)	—	Catterall (2011) CaVβ2 regulates cardiomyocytes hypertrophy
Brain	Episodic Ataxia, Type 5	CACNB4 (causal mutation)	Tsien et al. (1988); Zamponi et al. (2015)	—
	Epilepsy, Idiopathic generalized 9		Tsien et al. (1988)	—
	Epilepsy, Myoclonic Juvenile		Tsien et al. (1988)	Curtis and Catterall (1984) The human CACNB4 mutation prevents CaVβ4 to gets to the nucleus and modulates gene expression
Skeletal muscle	Epilepsy, Myoclonic Juvenile	CACNB1E (age-related decline of expression)	—	Tanabe et al. (1987) Restoration of CaVβ1E rescues GDF5 expression and prevents age-related skeletal muscle wasting

In 2017, the importance of *CACNB2* (gene coding for CaVβ2) as a genetic modifier of a Hypertrophic CardioMyopathy (HCM), in which the causal gene was *MYBPC3* (Myosin-Binding Protein C), has been described for the first time (Zhang et al., 2017). The authors hypothesized that the potential mechanism modifying disease phenotype was based on the attenuation of CaV-dependent Ca^2+^ current associated with *CACNB2* mutations. However, an additional possibility came out a few years later with a study that correlated the reduced cardiomyocyte hypertrophy to a CaV-independent CaVβ2 function (Pickel et al., 2021). CaVβ2 localization and activity in cardiomyocyte nuclei were shown to significantly regulate Calpain activity and Calpastatin expression (Pickel et al., 2021), a pro-hypertrophic protease and its inhibitor, respectively. Even though these events have not been explicitly linked to the reduction of cardiomyocyte hypertrophy, a correlation between the two might be hypothesized and would need to be further studied (Table 1).

Our recent work demonstrated the key role played by CaVβ1E in the context of age-related muscle wasting. We showed that CaVβ1E/GDF5 pathway counteracted the loss of muscle mass after denervation and that this signaling was defective in aged muscle fibers. Importantly, we overexpressed CaVβ1E in aged mouse muscles leading to increased GDF5 expression and activation of its signaling and therefore enabling the prevention of muscle mass loss and force decline during aging (Traoré et al., 2019). Importantly, the expression of an analogous of CaVβ1E has been also discovered in human muscle, decreasing in an age-related manner, indicating that the defective hCaVβ1E signaling might also be impaired in sarcopenic patients and suggesting the CaVβ1E/GDF5 axis having a therapeutic potential in muscle aging and linked pathologies (Traoré et al., 2019) (Table 1).

Nevertheless, little is known about the causes behind the decrease of CaVβ1E expression in aged muscles. One hypothesis we assessed, was a damaged neuro-muscular junction (NMJ), but we did not detect any NMJ changes in the 78-week old mice involved in the study which could have testified toward changes in electrical activity and modifications in basal CaVβ1E levels (Traoré et al., 2019). Chromatin methylation/demethylation events or other epigenetic alterations could further explain the unbalanced CaVβ1E/GDF5 axis in old muscle and should be investigated in future works.

To summarize, mutations in CaVβs encoding genes have currently been associated with disorders and, even if the pathological mechanisms have not always been fully characterized, both CaVβs roles linked or unlinked to CaVs can be argued. Brugada Syndrome type 4 and Episodic ataxia type 5 present causative mutations in genes coding for CaVβs proteins and other CaV subunits (CACNA2D1 and CACNA1A, and CACNA1A respectively–MalaCard database), corroborating CaVβ involvement in pathological mechanisms in a CaV-linked way (Table 1). Nevertheless, and as described above, CaVβ may also originate or modify pathological features independently of CaVs and an underestimation of these situations can be hypothesized.

## Conclusion

While CaVβs have long been considered to present exclusively CaVs’ linked functions, we depicted in this review the elements pointing towards a far more extensive significance in organ and cell homeostasis. CaVβs proteins have been described as efficient modulators of gene expression, either through their effect on Ca^2+^ signaling or through their DNA-related activities enabled by their nuclear localization. Regardless of the molecular mechanisms in which CaVβs are implicated, these proteins have been shown to influence cell and tissue capability to respond to different stimuli and to adapt following environmental changes. In the light of the findings of these two last decades, the impact of CaVβ1 and CaVβ4 on gene expression has been demonstrated several times, whereas CaVβ2 and CaVβ3 are less deeply characterized. A better establishment and appreciation of CaVβs relevance in cell biology will probably strengthen our understanding in a plethora of physiological and pathological mechanisms.
